# *Mycoplasma genitalium* infection among gay, bisexual and other men who have sex with men in Montréal, Canada

**DOI:** 10.14745/ccdr.v49i1112a03

**Published:** 2023-11-01

**Authors:** Anne-Sophie Lê, Annie-Claude Labbé, Alain Fourmigue, Milada Dvorakova, Joseph Cox, Claude Fortin, Irene Martin, Daniel Grace, Trevor Hart, David Moore, Gilles Lambert, the Engage Study Team

**Affiliations:** 1 Faculté de médecine, Université de Montréal, Montréal, QC; 2 Division of Infectious Diseases and Microbiology, Hôpital Maisonneuve-Rosemont, Centre intégré de santé et de services sociaux de l’Est-de-l’Île-de-Montréal, Montréal, QC; 3 Research Institute of the McGill University Health Centre, Montréal, QC; 4 Direction régionale de santé publique de Montréal, Centre intégré de santé et de services sociaux du Centre-Sud-de-l’Île-de-Montréal, Montréal, QC; 5 Division of Infectious Diseases and Microbiology, Centre hospitalier de l’Université de Montréal, Montréal, QC; 6 National Microbiology Laboratory, Public Health Agency of Canada, Winnipeg, MB; 7 Dalla Lana School of Public Health, University of Toronto, Toronto, ON; 8 Department of Psychology, Toronto Metropolitan University, Toronto, ON; 9 British Columbia Centre for Excellence in HIV/AIDS, Vancouver, BC; 10 Faculty of Medicine, University of British Columbia, Vancouver, BC; 11 Institut National de Santé Publique du Québec, Montréal, QC

**Keywords:** *Mycoplasma genitalium*, gbMSM, sexually transmitted infections, azithromycin, moxifloxacin, resistance

## Abstract

**Background:**

The bacteria *Mycoplasma genitalium* has been identified as a causative agent of urethritis in men, especially in gay, bisexual and other men who have sex with men (gbMSM). Canadian clinic-based data have identified a high prevalence of *M. genitalium* and resistance to antibiotic treatments. This article estimates the prevalence of *M. genitalium* infections among Montréal gbMSM, explores correlates for *M. genitalium* infection and estimates the prevalence of mutations associated with antimicrobial resistance (AMR).

**Methods:**

Engage Cohort Study is a multi-site longitudinal study on sexually active gbMSM, aged 16 years and older, recruited via respondent-driven sampling in Montréal, Toronto and Vancouver. Participants completed a questionnaire on behaviour and were tested for sexually transmitted and blood-borne infections at each visit. For this sub-study, Montréal participants with a follow-up visit that occurred between November 2018 and November 2019 were included.

**Results:**

A total of 2,064 samples were provided by 716 participants. Prevalence of *M. genitalium* infection was 5.7% at rectal and/or urethral sites, 4.0% at rectal site and 2.2% at urethral site. Correlates for *M. genitalium* infection were younger age and reporting six or more sexual partners in the past six months. Prevalence of macrolide resistance associated mutations (MRAM), quinolone resistance associated mutations (QRAM) and either MRAM or QRAM, was 82%, 29% and 85%, respectively.

**Conclusion:**

This first population-based study among gbMSM in Canada documents a high prevalence of urethral and rectal *M. genitalium* infection and high levels of AMR. Our results highlight the importance of access to testing and AMR detection when indicated.

## Introduction

*Mycoplasma genitalium* has been identified as a growing health concern for sexually active gay, bisexual and other men who have sex with men (gbMSM) by causing acute, persistent or recurrent urethritis ((1–6)). The data concerning *M. genitalium* as a causative agent of clinical proctitis are conflicting ((4–8)). *Mycoplasma genitalium* co-infection with other bacterial sexually transmitted infections (STIs) has been frequently reported in gbMSM ((7,9)).

*Mycoplasma genitalium* infection is not a notifiable condition in Canada ((10,11)) yet there are no published Canadian community-based studies concerning *M. genitalium* infection. Studies conducted in 2013 (Ontario), 2016 (Alberta) and 2019 (Saskatchewan), among men and women who had STI symptoms or sought medical attention for STI screening, have shown high rates of *M. genitalium* infection and macrolide resistance associated mutations (MRAM) and a significant presence of quinolone resistance associated mutations (QRAM) ((12–14)).

More detailed Canadian data are required to guide testing and treatment of *M. genitalium* infections in gbMSM. The objectives of this study are to 1) estimate the prevalence of *M. genitalium* infection and other selected bacterial STIs by anatomical site among Montréal gbMSM, 2) explore correlates of *M. genitalium* infection and 3) estimate the prevalence of MRAM and QRAM.

## Methods

### Engage Cohort Study

Engage Cohort Study is a collaboration between researchers and community-based organizations to study the sexual health, including human immunodeficiency virus (HIV) and sexually transmitted and blood-borne infections (STBBIs), of gbMSM in Montréal, Toronto and Vancouver. Details for this cohort study were described elsewhere ((15–17)). In brief, participants were recruited using respondent-driven sampling (RDS), a survey method for sampling hard-to-reach populations deriving from chain referral sampling ((18)). Thus, enrolled participants recruited other eligible participants through their social networks. Eligibility criteria were as follows: French or English-speaking cisgender or transgender men; 16 years of age or older; and reporting at least one sexual encounter with a man in the prior six months. After recruitment, participants were invited every 6–12 months for subsequent visits at the community study site. At each visit, participants completed a self-administered computer-assisted questionnaire and provided biological samples, including first-pass urine, a pharyngeal and a rectal swab and a blood sample.

### Sub-study in Montréal

Montréal recruitment into the Engage Cohort Study started in February 2017. For this one-time point sub-study, participants with a follow-up visit that occurred between November 2018 and November 2019 were included.

### Biological specimen collection and laboratory testing

To detect *Neisseria gonorrhoeae* and *Chlamydia trachomatis*, nucleic acid amplification tests were used (cobas^®^ 4800; Roche Diagnostics, Branchburg, New Jersey). For *M. genitalium* detection, samples were kept at room temperature in the cobas^®^ PCR Media (Roche Diagnostics) for a maximum of one year or as frozen eluates. Specimens were analyzed using the Allplex^TM^ CT/NG/MG/TV assay (Seegene Inc.). *Mycoplasma genitalium*-positive samples were subsequently analyzed by real-time PCR to detect MRAM and QRAM by using the Allplex^TM^ MG & AziR and Allplex^TM^ MG & MoxiR assays, respectively.

### Outcomes and correlates

Using current knowledge based on existing literature, variables were selected from the Engage Cohort Study questionnaire ((19,20)). Variables were grouped into the following categories: sociodemographic; sexual partners in the past six months (P6M); methods of finding sexual partners in the P6M; substance use in the P6M; and STBBIs in the P6M. The variable “chemsex” was defined as crystal methamphetamine, gamma-hydroxybutyrate (GBH), ecstasy/3,4-methylenedioxymethamphetamine (MDMA), ketamine, or poppers (i.e. alkyl nitrites) consumption in the two hours before or during sex with at least one of the last five sexual partners in the P6M. The variable “self-reported STI diagnosis” refers to a diagnosis by a healthcare professional in the P6M of *C. trachomatis*, *N. gonorrhoeae*, lymphogranuloma venereum (LGV) or syphilis. An individual was considered to have an *M. genitalium* infection if either their urine or their rectal sample was positive. Key mutations associated with azithromycin resistance (positions 2058 or 2059 in region V of the 23S ribosomal ribonucleic acid gene) and moxifloxacin resistance (S83I, S83R, S83N, D87N, or D87Y in *parC*) were used to define MRAM and QRAM, respectively.

### Statistical analyses

Prevalence and odds ratios (OR) were estimated and adjusted for the recruitment method as well as censoring, using a combination of RDS-II weights ((21)) and inverse-probability-of-censoring weights ((22)). The RDS-II weights are inversely proportional to the participants’ network size, meaning that data for individuals with large networks were weighted less. The 95% confidence intervals (CI) were calculated using robust (sandwich) variance estimation to account for the within-subject correlation induced by weighing ((23)). Prevalence data was not adjusted MRAM and QRAM since one individual with a larger weight could easily dominate the subsample within small subsamples (each MRAM and QRAM subsample had n fewer than 100 positive specimens). Logistic regression was used to predict *M. genitalium* infection among gbMSM. Since the aim was prediction, there was no need to consider confounding or effect modification. Predictive performance was assessed using Akaike information criterion (AIC).

### Ethics

Ethics approval was received from the Research Institute of the McGill University Health Centre.

## Results

**Figure 1:**
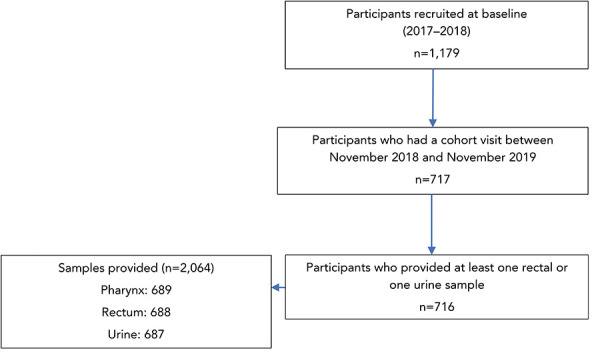
Flow diagram of Engage Cohort Study in Montréal study participants and samples included in the analysis, by anatomical sampling sites

Between February 2017 and June 2018, 1,179 participants were recruited in Montréal. A follow-up study visit, during which samples were collected for *M. genitalium* testing, occurred for 717 participants. One participant was excluded from *M. genitalium* prevalence analyses because only a pharyngeal sample was provided. Overall, 716 participants provided a total of 2,064 samples (**Figure 1**).

Most participants identified their ethnocultural identity as French or English Canadian (53.5%) and their sexual orientation as gay (82.2%). The majority reported having an education level higher than high school level (79.1%), a gross annual income of $30,000 or less (60.1%), being HIV-negative (84.9%) and having five or fewer male sexual partners in the P6M (67.6%) (**Table 1**).

**Table 1:** Sociodemographic characteristics of the Engage Cohort Study in Montréal participants^a^ who provided specimen(s) for *Mycoplasma genitalium* analysis, November 2018–November 2019, n=716

Characteristics	Adjusted proportion (%)^b^	95% CI
**Age (years)**		
29 or younger	30.2	24.5–36.7
30–45	38.5	31.6–45.8
46 or older	31.3	24.6–38.9
**Education level**		
High school degree or less	20.9	15.7–27.3
More than high school degree	79.1	72.7–84.3
**Annual income (CAD)**		
0–29,999	60.1	53.1–66.7
30,000–59,999	31.7	25.6–38.6
60,000 or more	8.2	6.1–11.0
**Ethnocultural group**		
French Canadian	45.0	37.9–52.4
English Canadian	8.5	5.6–12.5
European	12.7	9.1–17.4
Latin American	13.7	9.1–20.1
South or East Asian	4.9	2.1–11.2
Arab or North African	5.8	3.2–10.2
East or West African or Caribbean	3.5	1.7–7.1
Other^c^	5.9	3.4–10.1
**Immigration**		
Born in Canada	59.2	51.7–66.2
Moved to Canada in the past 2 years	5.1	2.7–9.3
Moved to Canada in the past 3 years or more	35.7	28.4–42.9
**Gender identity**		
Cisgender man	92.9	88.7–95.7
Transgender man	1.8	0.6–5.4
Other^d^	5.2	3.1–8.8
**Sexual orientation**		
Gay	82.2	76.6–86.7
Bisexual	9.2	6.0–13.8
Queer	4.6	2.6–8.2
Other^e^	4.0	2.1–7.3
**Sexual behaviours P6M**		
Any condomless anal sex	56.0	48.4–63.4
Any chemsex^f^	10.8	7.7–14.9
**Number of male sexual partners**		
5 or fewer	67.6	61.0–73.6
6–10	16.0	11.4–21.9
11 or more	16.4	12.5–21.2
**HIV status**		
Living with HIV	15.1	11.0–20.3

Abbreviations: CI, confidence interval; HIV, human immunodeficiency virus; P6M, past six months

^a^ Montréal’s Engage Cohort Study participants who had a follow-up visit in the window period between November 2018 and November 2019 and provided at least one rectal or urethral sample

^b^ Adjusted for respondent-driven sampling recruitment and censoring

^c^ Other ethnocultural group included Aboriginal or Indigenous

^d^ Other sexual orientations included bisexual and queer

^e^ Other gender identities included participants identifying as genderqueer, non-binary, or two-spirit

^f^ Chemsex includes crystal methamphetamine, GHB (gamma-hydroxybutyrate), ecstasy/MDMA (3,4-methylenedioxymethamphetamine), or ketamine consumption in the two hours before or during sex with at least one of the last five partners participants reported having sex within the P6M ((17)). Poppers (i.e. alkyl nitrites) are included in the chemsex definition

### Prevalence of *Mycoplasma genitalium* infection and other sexually transmitted infections

*Mycoplasma genitalium* prevalence was 5.7% (95% CI: 4.0–8.1) (rectal or urethral site) with anatomical site-specific prevalence being 4.0% (95% CI: 2.6–6.0) at the rectal site and 2.2% (95% CI: 1.2–4.0) at the urethral site (**Table 2**). The overall prevalence of *M. genitalium* was detected at the pharyngeal site in only two individuals (0.2%, 95% CI: 0.1–0.9). Prevalences of *C. trachomatis* and *N. gonorrhoeae* are detailed in Table 2. Among the individuals with urethral *C. trachomatis* infection, one of five were co-infected with *M. genitalium* (20%); among those with rectal *C. trachomatis* infection, two of 22 were co-infected with *M. genitalium* (9.1%) (**Table 3**). Among those with rectal *N. gonorrhoeae* infection, two of 12 were co-infected with *M. genitalium* (16.7%); no urethral *N. gonorrhoeae* infection was observed.

**Table 2:** Prevalence of *Mycoplasma genitalium*^a^ and of *Neisseria gonorrhoeae* and *Chlamydia trachomatis* infections^b^ by anatomical site, n=716

Type of sample (n)	Positive samples	Adjusted prevalence^c^	
	n	%	95% CI
**Pharyngeal swab (n=688)**			
*M. genitalium*	2	0.2	0.1–0.9
*N. gonorrhoeae*	15	1.5	0.8–2.6
*C. trachomatis*	7	0.8	0.2–3.0
**Urethral swab (n=687)**			
*M. genitalium*	23	2.2	1.2–4.0
*N. gonorrhoeae*	0	0.0	N/A
*C. trachomatis*	5	1.9	0.4–8.6
**Rectal swab (n=688)**			
*M. genitalium*	41	4.0	2.6–6.0
*N. gonorrhoeae*	12	1.4	0.6–3.3
*C. trachomatis*	22	2.6	1.2–5.5
**Rectal or urethral swab (n=716)**			
*M. genitalium*	61	5.7	4.0–8.1

Abbreviations: CI, confidence interval; *C. trachomatis*, *Chlamydia trachomatis*; *M. genitalium*, *Mycoplasma genitalium*; *N. gonorrhoeae, Neisseria gonorrhoeae*; N/A, not available

^a^ Using Allplex^TM^ CT/NG/MG/TV Assay

^b^ Using the cobas^®^ 4800 system

^c^ Adjusted for respondent-driven sampling recruitment and censoring

**Table 3:** Co-infections of *Mycoplasma genitalium*, *Neisseria gonorrhoeae* and *Chlamydia trachomatis* by anatomical site, n=716

Type of sample (n)		*C. trachomatis*		*N. gonorrhoeae*	
		Negative	Positive	Negative	Positive
**Urethral swab (n=672)**					
*M. genitalium*	Negative	645	4	649	0
	Positive	22	1	23	0
**Rectal swab (n=683)**					
*M. genitalium*	Negative	622	20	632	10

Positive

39

2

39

2

Abbreviations: *C. trachomatis*, *Chlamydia trachomatis*; *M. genitalium*, *Mycoplasma genitalium*; *N. gonorrhoeae*, *Neisseria gonorrhoeae*

### *Mycoplasma genitalium* infection correlates

Younger age (29 years or younger) and the following factors (all reported in the past six months) were significantly associated in univariate analysis having more male sexual partners (6–10 partners and 11 or more partners compared to five or fewer); having at least one new sexual partner; reporting at least one condomless anal sex act (insertive or receptive) with another man; engaging in chemsex; and having received a diagnosis of an STI (**Table 4**). Living with HIV was not associated with *M. genitalium* infection. The best predictive regression model of *M. genitalium* infection included the following factors: younger age (29 years or younger) (OR: 2.5, 95% CI: 1.2–5.5); and declaring more male sexual partners P6M (6–10 partners and 11 or more partners) (respective OR: 3.3, 95% CI: 1.3–8.5, and OR: 5.7, 95% CI: 2.3–14.1) (**Table 5**).

**Table 4:** Correlates of *Mycoplasma genitalium* infection (urethral or rectal site) in univariate analyses (n=716)

Characteristics	aOR^a^	95% CI
**Sociodemographics**		
**Age (years)**		
46 or older	Reference	
30–45	1.0	0.4–2.6
29 or younger	2.9	1.3–6.5
**Born in Canada**		
No	Reference	
Yes	1.0	0.5–1.9
**Ethnocultural group**		
French Canadian	Reference	
English Canadian	1.5	0.5–4.5
European	2.8	0.9–7.8
Latin American	0.5	0.1–2.0
South or East Asian	0.9	0.2–5.0
Other^b^	1.2	0.4–3.1
**Education level**		
Higher than high school degree	Reference	
High school degree or less	0.5	0.2–1.4
**Annual income (CAD)**		
0–29,999	Reference	
30,000–59,999	1.8	0.9–3.6
60,000 or more	2.9	1.0–7.5
**Sexual orientation**		
Gay	Reference	
Other^c^	0.6	0.2–1.7
**Gender identity**		
Cisgender man	Reference	
Transgender man	2.4	0.4–13.2
Other^d^	1.8	0.6–3.1
**Living with HIV**		
No	Reference	
Yes	1.4	0.6–3.1
**Sexual partners (P6M)**		
**Number of male sexual partners**		
5 or fewer	Reference	
6–10	3.9	1.5–10.3
11 or more	7.4	3.1–17.7
**New sex partner**		
No	Reference	
Yes	3.9	1.5–10
**Condomless anal sex acts with a man**		
None	Reference	
1 or more	3.3	1.3–8.6
**Methods of finding sexual partners (P6M)**		
**Attending a bath house or sex club**		
No	Reference	
Yes	1.4	0.7–2.8
**Attending a group sex event**		
No	Reference	
Yes	2.4	0.9–6.4
**Substance use (P6M)**		
**Any chemsex^e^**		
No	Reference	
Yes	2.3	1.2–4.4
**Crystal methamphetamine use**		
No	Reference	
Yes	2.0	0.8–4.9
**Drug injection**		
No		Reference
Yes	N/A^f^	N/A
**STBBI (P6M)**		
**Self-reported sexually transmitted infection diagnosis^g^**		
No	Reference	
Yes	3.3	1.4–7.9
**Co-infection with *C. trachomatis* or *N. gonorrhoeae***		
No	Reference	
Yes	1.4	0.4–3.6

Abbreviations: aOR, adjusted odds ratio; CI, confidence interval; *C. trachomatis*, *Chlamydia trachomatis*; *N. gonorrhoeae*, *Neisseria gonorrhoeae*; N/A, not applicable; P6M, past six months; STBBI, sexually transmitted and blood-borne infections

^a^ Adjusted for respondent-driven sampling recruitment and censoring

^b^ Other ethnocultural groups included Arab or North African, East or West African or Caribbean and Aboriginal or Indigenous

^c^ Other sexual orientations included bisexual and queer

^d^ Other gender identities included participants identifying as genderqueer, non-binary, or two-spirit

^e^ Chemsex includes crystal methamphetamine, GHB (gamma-hydroxybutyrate), ecstasy/MDMA (3,4-methylenedioxymethamphetamine) or ketamine consumption in the two hours before or during sex with at least one of the last five partners participants reported having sex within the P6M ((17)). Poppers (i.e. alkyl nitrites) are included here in chemsex definition

^f^ Too few *M. genitalium* infections among participants who injected drugs to permit valid inference

^g^ Self-reported STI (sexually transmitted infection) diagnosis by a healthcare professional in the P6M like *C. trachomatis*, *N. gonorrhoeae*, lymphogranuloma venereum (LGV) or syphilis

**Table 5:** Multivariable predictive model of *Mycoplasma genitalium* infection

Characteristics	aOR^a^	95% CI
**Number of male sexual partners P6M**		
5 or fewer	Reference	
6–10	3.3	1.3–8.5
11 or more	5.7	2.3–14.1
**Age (years)**		
30 or older	Reference	
29 or younger	2.5	1.2–5.5
**Condomless anal sex at least once P6M**		
No	Reference	
Yes	2.1	0.8–5.4

Abbreviations: aOR, adjusted odds ratio; CI, confidence interval; *M. genitalium*, *Mycoplasma genitalium*; P6M, past six months

^a^ Adjusted for respondent-driven sampling recruitment and censoring

### Antimicrobial resistance of *Mycoplasma genitalium*

For the three participants who were infected at both the urethral and rectal sites, the results obtained from the urethral site were used to calculate the prevalence of antimicrobial resistance (AMR). Prevalence of MRAM was 82% (n=46/56) and prevalence of QRAM was 29% (n=16/55) (**Table 6**). Prevalence of either MRAM or QRAM was 85% (n=46/54), while prevalence of both MRAM and QRAM was 28% (n=15/54).

**Table 6:** Macrolide resistance and quinolone resistance-associated mutations detected by real-time polymerase chain reaction in *Mycoplasma genitalium*-positive specimens, n=61^a^

Resistance-associated mutations (genes)	Mutations	Real-time polymerase chain reaction results	
		n	%
MRAM (23S rRNA), n=56^b^	Wild type	10	18%
	A2058G	7	12%
	A2059G	39	70%
QRAM (*parC*^c^), n=55^b^	Wild type	39	71%
	S83I (G248T)	13	23%
	S83R (A247C)	2	4%
	D87Y (G259T)	1	2%

Abbreviations: *M. genitalium*, *Mycoplasma genitalium*; MRAM, macrolide resistance-associated mutations; QRAM, quinolone resistance-associated mutations; rRNA, ribosomal ribonucleic acid

^a^ The 64 positive samples obtained from the urethral or rectal site were from 61 distinct individuals (three were infected at both sites). For the three participants who were infected at both the urethral and rectal site, the results obtained from the urethral site were used to calculate the prevalence of resistance

^b^ An invalid result (amplification failure) was obtained in five cases for the macrolide assay and six cases for the quinolone assay

^c^ No mutation in the *gyrA* gene, nor the S83N or the D87N mutations in *parC* was found

## Discussion

This first Canadian community-based study estimates the prevalence of *M. genitalium* infection at 5.7% (urethral or rectal infection) among gbMSM. It is challenging to contextualize our data since population-based prevalence studies are lacking. Compared to Canadian STI clinic-based studies, the urethral *M. genitalium* prevalence in our study (2.2%) was lower than previous estimates among men in Ontario (4.5%, 2013), Alberta (5.3%, 2016) and Saskatchewan (6.2%, 2019) ((12–14)). In Australia, urethral *M. genitalium* prevalence among men who have sex with men (MSM) recruited in STI clinics ranged from 2.7–4.7% and prevalence of rectal infections (7.0%–8.9%) was higher than in our study (4.0%) ((24,25)). Consistent with our results (n=3/689; 0.4%), a very low number of pharyngeal *M. genitalium* infections among MSM were reported in Australia (n=0/508 to n=8/464; 2.0%) ((9,25)). We hypothesize that oral transmission is negligible, and we excluded *M. genitalium*-positive pharyngeal samples from our prevalence estimation. Rectal *M. genitalium* infection (4.0%) was more common than rectal *C. trachomatis* (2.6%) and *N. gonorrhoeae* (1.4%) infections. Urethral *M. genitalium* prevalence estimates were more similar to those of *C. trachomatis* infection (*M. genitalium*, 2.2%; *C. trachomatis*, 1,9%; *N. gonorrhoeae*, 0%). A United States cohort study conducted in 2018–2019 among young gbMSM and transgender women found that *M. genitalium* was more prevalent than other STIs in both rectal (*M. genitalium*, 21.7%; *C. trachomatis*, 8.8%; *N. gonorrhoeae*, 6.8%) and urine samples (*M. genitalium*, 8.9%; *C. trachomatis*, 1.6%; *N. gonorrhoeae*, 0.8%) ((26)). A 2017–2018 Australian study found that among asymptomatic MSM, *C. trachomatis* prevalence was comparable to *M. genitalium* in rectal samples (*M. genitalium*, 7.0%; *C. trachomatis,* 8.5%; *N. gonorrhoeae*, 6.2%) and urine samples (*M. genitalium*, 2.7%; *C. trachomatis*, 1.7%; *N. gonorrhoeae*, 0.7%). It also found that 9.2% of MSM that tested positive for rectal *C. trachomatis* were co-infected with *M. genitalium* while 6.1% of positive rectal *N. gonorrhoeae* samples demonstrated co-infection with *M. genitalium* ((24)). In our study, 9.1% of gbMSM that tested positive for *C. trachomatis* at the rectum were co-infected with *M. genitalium*, 16.7% of rectal *N. gonorrhoeae* infections showcased *M. genitalium* co-infection.

In univariate analyses, multiple risk factors for STI transmission, such as chemsex P6M, new sexual partners P6M and a STI diagnosis P6M, were identified. Younger age and having multiple male sexual partners were retained in our predictive model. These findings are consistent with studies that identified younger age ((24,27,28)) and multiple sexual partners ((19,20,29,30)) as correlates of *M. genitalium* infection. While a United Kingdom study documented a higher prevalence of *M. genitalium* among gbMSM living with HIV ((31)), HIV infection was not associated with *M. genitalium* infection in our study. More studies are needed to clarify the role of *M. genitalium* in HIV acquisition or transmission among gbMSM as it has been identified as a risk factor of HIV infection, especially in MSM ((32,33)).

The very high prevalence of MRAM (82%; n=46/56) and QRAM (29%; n=16/55) found among the Engage Cohort Study Montréal’s gbMSM is a worrisome finding. This prevalence is higher than previous Canadian MRAM estimates (men in Alberta in 2016, 64%; women and men in Saskatchewan in 2019, 63% and men in Ontario in 2013, 63%) ((12–14)). Treatment failure with azithromycin has been well described with single nucleotide polymorphisms at positions 2058 and 2059 in region V of the 23S ribosomal ribonucleic acid ((34)). For QRAM, S83 in the *parC* gene is significantly associated with moxifloxacin resistance ((34)). While several single nucleotide polymorphisms contribute to quinolone resistance, none are as strong predictors of treatment failure than macrolide resistance with 23S ribosomal ribonucleic acid single nucleotide polymorphisms ((34,35)). Previous Canadian studies found a QRAM prevalence of 11%–20% among men and women ((12–14)). A meta-analysis compiling studies from 2010–2019 estimated MRAM and QRAM prevalence at 52% and 10%, respectively, in the Americas region ((2)). A 2017–2018 United States clinic-based study among men with urethritis found MRAM and *parC* QRAM prevalence levels of 64% and 12%, respectively ((28)). Being infected with a macrolide-resistant *M. genitalium* is more likely in gbMSM than in women and men with female partners only ((1,36,37)). This could be explained by transmission in closely-knit sexual networks and increased exposure to antibiotics ((37)). The increasing azithromycin resistance could be explained by its widespread use for the treatment of certain STIs ((2,7,38–40)). In our study, 28% of *M. genitalium*-positive samples had both MRAM and QRAM. Dual resistance has already been reported in gbMSM on HIV PrEP and those living with HIV ((36,41)).

### Implications for research and practice

In our study, we identified a high prevalence of *M. genitalium* infections among gbMSM, especially among younger individuals and those reporting multiple male sexual partners. Although most current guidelines state that routine screening for *M. genitalium* infection is not recommended (as it would contribute to selection pressure of resistant strains), they vary in terms of testing indications and timing in symptomatic individuals ((42–44)): at the time of initial presentation of urethritis (concomitantly with *N. gonorrhoeae* and *C. trachomatis* testing) ((42–44)), only for recurrent non-gonococcal urethritis ((4)) or only for non-chlamydial non-gonococcal persistent or recurrent urethritis, following empiric treatment for *N. gonorrhoeae* and *C. trachomatis* and when pretreatment nucleic acid amplification tests or follow-up test of cure are negative for *C. trachomatis* and *N. gonorrhoeae* ((45)). Regarding rectal screening, some clearly state it is not recommended ((4)) or do not mention extra genital testing ((46)). The high prevalence of *M. genitalium* infection among gbMSM with *C. trachomatis* or *N. gonorrhoeae* infection demonstrates the need for clinicians to remain highly vigilant of a possible co-infection in the case of persistent symptoms after adequate treatment. Our findings of 4.0% prevalence of rectal *M. genitalium* among gbMSM in Montréal, being almost two-fold the prevalence of urethral *M. genitalium* infection (2.2%), and much higher than *N. gonorrhoeae* rectal infection (1.4%) or *C. trachomatis* infection (2.6%), may add to epidemiologic evidence in the process of updating the Canadian guidelines ((45)). Finally, the most recent guidelines touching upon the management of *M. genitalium* infection recommend AMR-guided therapy ((4,42,44)). This approach has demonstrated potential in reducing treatment failures ((47,48)). Based on the identified susceptibility profile, doxycycline is used as initial empiric treatment and is followed by either azithromycin or moxifloxacin ((49)). Because of limited availability of tests in Canada and according to the current Canadian guidelines, treatment initiation for *M. genitalium* should occur in the context of syndromic management of persistent or recurrent urethritis ((10)). Recommended treatment consists of azithromycin and moxifloxacin as first and second lines of treatment ((45)). The high AMR observed in our study supports the need for *M. genitalium* detection and AMR testing in a short turnaround time ((42,44,47)). It also highlights the need, when both QRAM and MRAM are detected, for an easier and quicker access to alternative treatments such as pristinamycin, which can currently be requested through the Health Canada’s special access program ((42,46,50)).

## Limitations

The small sample size limited our ability to identify correlates of infection or AMR. Data regarding STI-related symptoms was not collected in the study questionnaire which was designed prior to the initiation of this sub-study and was focused on societal and community contexts, social relationships and sexual behaviour. Hence, we could not evaluate the prevalence of *M. genitalium* in association with clinical presentation. Despite using the RDS method for recruitment, some subgroups of the gbMSM population may be over- or under-represented. Potential biases related to RDS were attenuated by adhering to recommended recruitment procedures, having a large sample size with long recruitment chains and adjusting with RDS-II weights. The AMR data were not RDS-adjusted because they were obtained from too small a subsample. Our prevalence findings might not be generalizable to non-urban Canadian gbMSM populations. We did not find comparison studies analyzing the performance of the Allplex CT/NG/MG/TV Assay, which limited our appreciation of potential information biases. Le Roy *et al.* calculated an overall agreement of 94.6% between in-house real-time PCR and the Allplex MG & AziR Assay ((51)). The assay, however, showed low sensitivity for macrolide resistance compared to sequencing (sensitivity of 74.5%, specificity of 97.6%).

## Conclusion

This first population-based study among Canadian gbMSM documented a high prevalence of urethral and rectal *M. genitalium* infection. The observed levels of AMR, which exceed the 5% threshold at which a change in empirical treatment is recommended by the World Health Organization, supports the need for AMR-guided therapy ((52)). Efforts should be made to facilitate targeted *M. genitalium* detection and AMR testing when indicated.
